# Fructose, Omega 3 Fatty Acids, and Vitamin E: Involvement in Pediatric Non-Alcoholic Fatty Liver Disease

**DOI:** 10.3390/nu12113531

**Published:** 2020-11-17

**Authors:** Gigliola Alberti, Juan Cristóbal Gana, José L. Santos

**Affiliations:** 1 Gastroenterology and Nutrition Department, Division of Pediatrics, School of Medicine, Pontificia Universidad Católica de Chile, Santiago 3580000, Chile; galberti@med.puc.cl (G.A.); jcgana@gmail.com (J.C.G.); 2 Department of Nutrition, Diabetes and Metabolism, School of Medicine, Pontificia Universidad Católica de Chile, Santiago 3580000, Chile

**Keywords:** nonalcoholic fatty liver disease (NAFLD), NASH, nutrition, steatosis, sugar, fructose, omega 3 fatty acids, vitamin E

## Abstract

Non-alcoholic fatty liver disease (NAFLD) is currently the most common form of liver disease in both adults and children, becoming the leading cause for liver transplant in many countries. Its prevalence has increased considerably in recent years, mainly due to the explosive increase in pediatric obesity rates. NAFLD is strongly associated with central obesity, diabetes, dyslipidemia and insulin resistance, and it has been considered as the hepatic manifestation of the metabolic syndrome. Its complex pathophysiology involves a series of metabolic, inflammatory and oxidative stress processes, among others. Given the sharp increase in the prevalence of NAFLD and the lack of an appropriate pharmacological approach, it is crucial to consider the prevention/management of the disease based on lifestyle modifications such as the adoption of a healthy nutrition pattern. Herein, we review the literature and discuss the role of three key nutrients involved in pediatric NAFLD: fructose and its participation in metabolism, Omega-3 fatty acids and its anti-inflammatory effects and vitamin E and its action on oxidative stress.

## 1. Introduction

Non-alcoholic fatty liver disease (NAFLD) or, according to the new proposal definition, metabolic dysfunction-associated fatty liver disease (MAFLD) [1] is the most common form of liver disease in both adults and children [2] associated with the recent global obesity epidemic. NAFLD is a general term used to describe a broad range of liver abnormalities. The spectrum of conditions related to NAFLD includes benign macro vesicular hepatic steatosis, non-alcoholic steatohepatitis, hepatic fibrosis, cirrhosis of the liver, and hepatocellular carcinoma. The progression from steatosis to advanced liver disease and cirrhosis has been estimated to increase by up to 30% per decade in the adult population [3]. A complex set of interactions between genetic, epigenetic, and environmental factors are involved in the development and progression of pediatric NAFLD leading to the adult conditions [4]. NAFLD and its subtype non-alcoholic steatohepatitis (NASH) affect approximately 30% and 5% of the general population, respectively [5]. In the United States, 65 to 100 million individuals are estimated to have NAFLD [2,6]. Epidemiologic data derived from pediatric studies using non-invasive and invasive tests to diagnose NAFLD, indicate a 5% to 10% prevalence in the general pediatric population, increasing to 40% in obese or overweight children [7,8,9]. The highest rate in pediatric populations are the Hispanics, from 12% in general and up to 52% in obese adolescents [7]. Focusing on early diagnosis and characterization of NAFLD at an early stage is critical to define strategies to prevent the development of liver fibrosis, cirrhosis, and hepatocellular carcinoma, the leading causes of liver transplantation in adults. It is essential to increase the awareness of the medical community and health professionals of the public health dimensions of this problem and to identify current gaps to provide future directions for interventional research on the prevention and management of NAFLD.

There is a strong correlation between NAFLD and central obesity, diabetes, dyslipidemia and insulin resistance, and it is being considered as a manifestation of the metabolic syndrome (MS) affecting the liver [10,11]. Close to 90% of patients with NAFLD have at least one of the components of MS, and ~30% meet the full criteria for this diagnosis [12]. In a study of 197 overweight/obese white children (aged 3–19 years) with biopsy-proven NAFLD/NASH, increased abdominal circumference rather than body mass index (BMI) was found to be a better predictor of presence and severity of NASH [13]. The presence of insulin resistance (IR), type 2 diabetes mellitus (T2DM), hypertension, and other features of the metabolic syndrome also correlate well with the severity of the disease [10,14,15]. Among patients with T2DM, NAFLD has a prevalence of 70% [16] and it is well known that patients with diabetes had progressive fibrosis and cirrhosis of the liver [17]. The presence of both T2DM and NAFLD increases the likelihood of developing macro- and micro- vascular diabetes complications [17]. A meta-analysis of NAFLD and the risk of incident T2DM concludes that patients with NAFLD had a greater risk of incident diabetes than those without the disease (random-effects hazard ratio (HR) 2.22, 95% CI 1.84–2.60; *I*^2^ = 79.2%) [18].

Several studies have shown a higher incidence and prevalence of cardiovascular disease in patients with NAFLD, considering a possible role of NAFLD in the development of early atherosclerosis. Thus, these patients had an increased carotid wall intima-media thickness, atherosclerotic plaque and also increased plasma markers of endothelial dysfunction, independent of the degree of obesity [19,20,21]. These findings support the idea that altered metabolic markers in NAFLD-affected children translate to a parallel increase in adverse cardiovascular outcomes.

NAFLD is associated with higher risks of morbidity and mortality compared with age and sex matched groups. Pediatric patients have a 13.8 fold higher risk of dying or requiring liver transplantation due to liver cirrhosis when compared with the general population, based on a retrospective longitudinal study of 66 pediatric NAFLD patients followed for 20 years [22]. In a systematic review in adults, the risk of liver-related mortality increased exponentially with each increase in the stage of fibrosis: stage 1, mortality rate ratios (MMRs) = 1.41 (95% CI 0.17–11.95); stage 2, MRR = 9.57 (95% CI 1.67–54.93); stage 3, MRR = 16.69 (95% CI 2.92–95.36); and stage 4, MRR = 42.30 (95% CI 3.51–510.34) [23]. Furthermore, a higher risk of hepatocellular carcinoma was observed in adult NAFLD patients, regardless of whether NAFLD was associated to cirrhosis [24].

NAFLD is currently the leading cause for liver transplant in adults in many countries [24,25,26]. Furthermore, NAFLD represents the second cause of hepatocellular carcinoma leading to liver transplantation and it is currently the fastest rising indication for liver transplantation in patients with hepatocellular carcinoma [25,27]. The waiting list for liver transplant has been growing consistently over the past decade and the disparity between the organ donation and the increasing need for liver transplants has a direct influence on the mortality of these patients. An adequate strategy to diagnose NAFLD and prevent the progression of the liver disease are key features that will influence the morbidity and mortality of the patients, the liver transplant waiting list, and health resources.

The economic modeled prevalence of NAFLD in the United States using five prevalence models (one from United States and four from European countries) estimating 64 million people with NAFLD and considering the analysis of the Medicare database with the inpatient and outpatients cost for patients with NAFLD [28,29], shows that the annual direct medical cost is about $103 billion ($1613 per patient) [30]. This estimation includes all the different stages of the spectrum of the disease: from NAFLD to cirrhosis, liver transplantation, and death. In the other four European countries modeled, calculating 52 million people with NAFLD, the annual cost is about €35 billion (from €354 to €1163 per patient) [31,32].

The management of pediatric NAFLD is based on lifestyle modification, attainable through healthy weight reduction via diet and physical activity [33]. Studies conducted on the pediatric population that evaluate lifestyle dietary changes and weight loss have shown that interventions that result in persistent weight loss are associated with improvement in transaminases levels, liver echogenicity, and liver histology [34,35]. The 2017 North American Society for Pediatric Gastroenterology, Hepatology and Nutrition (NASPGHAN) NAFLD guideline [36] indicates that lifestyle modifications to improve diet and increase physical activity are recommended as the first line treatment, along with increasing moderate to high-intensity physical activity and limiting screen time activities to <2 h per day. No current medications are recommended to treat pediatric NAFLD because none have been proven to benefit the majority of patients [37,38,39,40,41,42,43].

The NAFLD pathophysiology is very complex and involves a number of metabolic processes, inflammation, and oxidative stress. In this line, different nutrients that may influence the natural history and progression of NAFLD have been studied, but the three mains ones in clinical trials have been fructose, Omega 3 fatty acids, and Vitamin E, in different pathophysiological mechanisms. The high consumption of fructose is involved in the activation of lipogenesis and the inhibition of fatty acid oxidation, promoting the development of NAFLD. Furthermore, low consumption of n-3 LCPUFAs and high n-6/n-3 ratios are described as a risk factor for NAFLD, at the same time that Vitamin E has been proposed as a therapeutic tool considering its antioxidant effect. With the aim of improving the understanding of the influence of these key nutrients on metabolic, inflammatory and oxidative processes, we carried out a bibliographic review to clarify the effects of fructose, omega 3 and vitamin E on pediatric NAFLD. We searched for original articles and reviews focusing on pediatric NAFLD nutritional treatment in PubMed and MEDLINE using the following search terms (or combination of terms): “NAFLD”, “NASH”, “fatty liver”, “macronutrient”, “dietary”, “recommendations”, “vitamin E”, “Omega 3”, “fatty acids”, “sugar”, and “fructose”. More weight was given to studies with a high level of evidence from randomized controlled trials, prospective case-control studies, meta-analyses or systematic reviews. We used reference lists of retrieved articles to obtain additional references.

## 2. Effects of Fructose, Omega-3 Fatty Acids and Vitamin E on Pediatric NAFLD

### 2.1. Reduction in Free Sugars and Sugar-Sweetened Beverages (SSBs)

The term “free sugar” refers to “all monosaccharides/disaccharides added to foods/beverages by the manufacturer/cook/consumer, plus sugars naturally present in honey/syrups/unsweetened fruit juices and fruit juice concentrates” [44]. Then, free sugars are mostly glucose, fructose, sucrose (glucose and fructose joined by glycosidic bond), and mixtures of glucose: fructose found in honey (approximately 50:50) or in high-fructose corn syrup (HFCS; 42% or 55% of fructose, named HFCS42 or HFS55, respectively). Consequently, sugars that are naturally contained in intact fruits (glucose, fructose, sucrose, and others) and in milk (lactose: glucose plus galactose joined by a glycosidic bond) are not considered as free sugars under this definition. On the other hand, added sugars include “all sugars used as ingredients in processed and prepared foods and sugars eaten separately or added to foods at the table” [45]. As a nutrient, glucose is present in foods as unbound glucose (e.g., in grapes, honey and HFCS), as part of disaccharides/oligosaccharides (e.g., sucrose, lactose and raffinose), attached to other dietary compounds (e.g., bound to the aglycone part of many dietary polyphenols) or being the monomer of polysaccharides of plant or animal origin (e.g., starch and glycogen). On the other hand, fructose is mainly found in food as sucrose, as unbound fructose (as it occurs in fruits, honey and HFCS) and as a monomer of undigestible fructo-oligosaccharides and fructans (e.g., inulin). Table sugar (sucrose) has been traditionally the most representative ingredient used as sweetener. From the 1970s and onwards, the use of the inexpensive industrial conversion of glucose from cornstarch to fructose has led the common consumption of processed foods that contain fructose as an additive, such as candy or desserts, and in sugar-sweetened beverages (SSBs) [46]. It has been reported that the intake of free sugars in children may contribute to 18.5% of total calories, being SSB the major single contributor in this category [45]. The high sweetness and hedonic value of fructose [47], coupled with a lower capacity of satiety of liquid sugars in SSB compared to equal amounts in solid foods, has contributed to its increased consumption in modern societies [48]. In children and adolescents, reasons for increased consumption of SSB includes an array of motivations including interpersonal relation issues, perceived physical/cognitive benefits, sensory/palatability properties, and external cues [49].

Given the deleterious effect of excessive sugar consumption on chronic diseases, the World Health Organization and the 2015–2020 Dietary Guidelines for Americans strongly recommend a reduction in the intake of free sugars to <10% of total energy intake [50,51], with an additional conditional recommendation of a further reduction to <5% [50]. This recommendation applies to adults; adolescents and children aged ≥2 years. Statements from the American Heart Association (AHA) and the European Society for Pediatric Gastroenterology, Hepatology and Nutrition (ESPGHAN) also adhere restriction in sugars specifically during childhood [44,45]. Even higher restriction has been proposed for infants and toddlers <2 years old [44,52].

**Figure 1:**
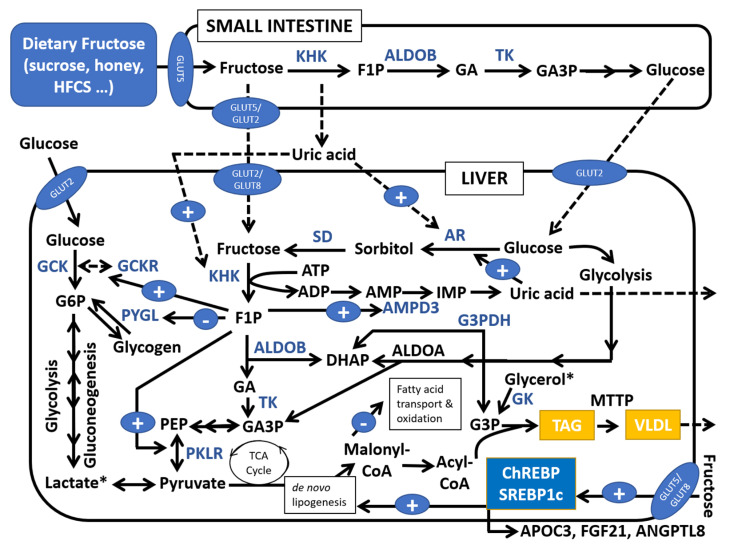
Metabolic fate of fructose in the liver after fructose ingestion. KHK: ketohexokinase (fructokinase); ALDOB: aldolase B; ALDOA: aldolase A; TK: triose kinase; GLUT2, 5 and 8: glucose-transporter 2, 5 and 8; SD: sorbitol dehydrogenase; AR: aldose reductase; ATP: adenosine triphosphate; ADP: adenosine triphosphate; AMP: adenosine monophosphate; IMP: inosine monophosphate; F1P: Fructose-1-phosphate; MTTP: microsomal triglyceride transfer protein; PKLR: pyruvate kinase, liver and red blood cell isoform; GA3P: glyceraldehyde 3-phosphate; TK: triose-kinase (also known as dihydroxyacetone kinase 2 or DAK); GA: glyceraldehyde; PEP: phosphoenolpyruvate; TAG: triacylglycerol; G6P: glucose-6-phosphate; GCK: glucokinase; GCKR: GCK regulatory protein; TCA: tricarboxylic acid; G3PDH: Glycerol-3-phosphate dehydrogenase; GK: glycerol kinase; AMPD3: adenosine deaminase; PYGL: Glycogen phosphorylase L; ChREBP: Carbohydrate-Responsive Element–Binding Protein; SREBP1c: Sterol Regulatory Element–Binding Protein 1c; APOC3: apolipoprotein C3; FGF21: Fibroblast Growth Factor-21; ANGPTL8: angiopoietin-like 8.

Both glucose and fructose are non-essential nutrients that can be synthetized in humans: glucose from gluconeogenic precursors in liver and, at a lesser extent, in kidney, and fructose from glucose through the polyol pathway. Glucose is only considered as a conditionally essential nutrient in rare genetic diseases affecting endogenous glucose production, such as glycogen storage disease type I [53]. Glucose is physiologically crucial since it is the center of a homeostatic neuroendocrine system that controls the blood levels of this monosaccharide across the feed-fasting cycle, which is disrupted in hyperglycemia of diabetes. On the other hand, fructose is only a crucial cellular nutrient as the primary energy source of sperm [54]. Although glucose and fructose are similar molecules, they differ in their transport and metabolism, both in the small intestine and in the liver. In the liver, a description of glucose metabolism in comparison to that of fructose is extensively described by Lustig et al. [55]. After ingestion of fructose, this monosaccharide is rapidly absorbed in enterocytes via GLUT5 (apical membrane) and GLUT5/GLUT2 (basolateral membrane), entering in the liver through GLUT2/GLUT8 transporters [54] (Figure 1). Fasting plasma levels of fructose are very low (10–50-fold lower than glucose), and even a large ingestion of this monosaccharide leads to small increases in its circulating levels (reaching 3–11 mg/dL), given that most fructose (>85%) is cleared in the splanchnic extraction [56]. It is assumed that fructose is mainly metabolized in the liver [54], although a recent report using isotope tracers in mice challenges this idea, also indicating a critical role of small intestine in fructose metabolism [57]. However, the shorter relative size of small intestine in humans versus mice still suggests a greater importance of liver versus intestine in fructose metabolism in humans [58]. In the liver, fructose is poorly metabolized by glucokinase (GCK), being hepatic fructose oxidation mainly driven by the action of ketohexokinase (KHK or fructokinase). KHK starts fructose degradation, leading to a faster metabolization of fructose compared to glucose, possibly due to the less-regulated steps of fructolysis compared to the highly regulated phosphorylation steps of glycolysis [59,60]. Production of 3-carbon metabolites of fructose by ALDOB (glyceraldehyde and dihydroxyacetone-P) are in equilibrium with glycerol 3-phosphate synthesis and de novo fatty acid synthesis that will be used in triglyceride generation [61]. Meanwhile, fructose in the liver suppresses fatty acid oxidation via inhibition of malonyl-CoA on fatty acid transport to mitochondria [59,62]. Enhanced de novo lipogenesis is amplified by the induction by fructose of Carbohydrate-Responsive Element–Binding Protein (ChREBP; also stimulated by glucose) and Sterol Regulatory Element–Binding Protein 1c (SREBP1c; mainly stimulated by insulin) [54]. Additionally, fructose-1P, the product of KHK reaction, indirectly induces the use and phosphorylation of glucose in the liver via interaction with the GCK regulatory protein, leading to accelerated glycogen synthesis. Fructose degradation is also related to rapid Pi and ATP depletion due to phosphorylation of glucose by KHK, which increases uric acid production by activation of AMP catabolism. Uric acid has been proposed as a key element linked to mitochondrial oxidative stress and fat accumulation [63]. Compared to glucose, fructose also seems to activate a higher secretion of the hepatokyne Fibroblast-Growth-Factor-21 (FGF21) involved in the regulation of insulin sensitivity, intrahepatic lipid accumulation and macronutrient selection [64]. Given all the above, it is concluded that high consumption of fructose is involved in the activation of de novo lipogenesis, inhibition of fatty acid oxidation, hepatic acceleration of glycogen synthesis, promotion of insulin resistance, hyperuricemia, increased postprandial lipemia, and development of NAFLD [54,56,58,65].

Interestingly, fructose also has an endogenous biosynthetic pathway initiated by the conversion of glucose to sorbitol catalyzed by the NADPH-dependent enzyme aldose reductase (AR), followed by the action of NAD-dependent sorbitol dehydrogenase (Figure 1). This pathway is believed to be a minor metabolic fate for glucose except in hyperglycemic status, where it is linked to diabetes complications [66]. It has been recently reported that fructose-induced hyperuricemia upregulates KHK and simultaneously activates AR, leading to increased endogenous fructose synthesis and leading to excessive intrahepatic accumulation [67]. In this context, studies conducted in children and adolescents indicate that dietary fructose intake and plasma uric acid have a relevant impact in NAFLD at pediatric ages [68,69,70]. Then, both synthetic and natural/dietary AR inhibitors may arise as potential agents against NAFLD [71].

In contrast to the negative views of dietary fructose on obesity-related chronic diseases [72], there are other more positive reports stating that dietary fructose is not more metabolically harmful in comparison to glucose [73], being both essentially providers of “empty calories”. As glucose and fructose are energetically matched and found together either in sucrose and HFCS, it is difficult to disentangle their separate effects on NAFLD and chronic diseases in human studies [74]. It was recently reported that isocaloric substitution with glucose and fructose represents no major differences on plasma glucose, insulin or plasma triglycerides [75] with expected differences of both sugars regarding postprandial plasma glucose and insulin, given the low glycemic index of fructose [76,77]. In a systematic meta-analysis of prospective studies, the association between SSB intake with increased metabolic syndrome risk was not extended to other important sources of dietary fructose [78]. Additionally, excessive weight gain in children is promoted by SSBs, which seems to be induced by its high caloric content [79,80]. Although Wehmeyer et al. [81] found that dietary energy excess is the primary factor associated with NAFLD, the unique features of fructose metabolism in the liver suggests a role of fructose intake in intrahepatic fat accumulation, as well as in the development of NAFLD and NASH [82,83,84,85,86]. Importantly, short-term isocaloric fructose restriction was associated with decreased liver fat, visceral fat, de novo lipogenesis and differential insulin kinetics in children with obesity [87]. Jin et al. [88] conducted a four-week randomized, controlled, double-blinded beverage intervention study in 24 overweight adolescents who had hepatic fat >8% on imaging, using calorie-matched study-provided fructose only or glucose only beverages. At the end of the study, the authors reported no significant change in hepatic fat or body weight in either group, but there was a significantly improved adipose insulin sensitivity, high sensitivity C-reactive protein, and low-density lipoprotein oxidation in the glucose beverage group. It has been recently reported in children with NAFLD that a diet low in carbohydrates and sugars may be more effective in reducing intrahepatic fat accumulation, insulin resistance and visceral fat compared to fat-restricted diets [89]. Therefore, it is envisaged that restriction of carbohydrates, fructose, free sugars or SSB are adequate interventions to prevent NAFLD in children [68,90,91,92].

The possible effect of dietary fructose on NAFLD through the microbiota also deserves a special mention. Studies in mice suggest that fructose in the small intestine is related to the development of endotoxemia, increasing gut-derived bacteria elements such as lipopolysaccharides and unmethylated DNA [93,94]. Such bacterial products are ligands of endogenous Toll-like receptor 4 (TLR4) triggering inflammation responses involved in NAFLD, NASH and liver fibrosis. It has been proposed that TLR4 may mediate the deleterious effects of dietary fructose on NAFLD, as demonstrated using TLR4-deficient mice [93]. Additionally, controlled studies involving either fructose or glucose challenges in adolescents (under both acute and chronic schemes) indicate higher postprandial endotoxin levels after fructose consumption compared to glucose [95,96]. Since reductions in sugars consumption are often accompanied by increased intake of natural or artificial sweeteners, there is a potential effect of such compounds on microbiota composition and subsequent effects on insulin resistance and NAFLD [97].

### 2.2. Omega-3 Long-Chain Polyunsaturated Fatty Acids

Polyunsaturated fatty acids (PUFAs) linoleic (omega-6 or n-6; 18:2Δ9, 12; LA) and α-linolenic (omega-3 or-3n; 18:2; Δ9,12, 15; ALA) are 18-carbon essential fatty acids in human diet. Desaturases (FADS1 and FADS2) and elongases (genes of the ELOVL family) acting on LA and ALA lead on one hand to the production of the n-6 long-chain polyunsaturated fatty acids (LCPUFA) arachidonic acid (AA; n-6; 20:4; among others; synthetized from LA) and, on the other hand, to the n-3 LCPUFAs eicosapentaenoic acid (EPA; n-3; 20:5) and docosahexaenoic acid (DHA; n-3; 22:6) (among others; both synthetized from ALA). AA is involved in the production of pro-inflammatory eicosanoids implicated in NAFLD development (2-series prostaglandins, 2-series tromboxanes and 4-series leukotrienes) while n-3 derivatives from EPA and DHA (3-series prostaglandins, 3-series tromboxanes, 5-series leukotrienes, as well as resolvins and protectins) are mildly inflammatory or anti-inflammatory, being also involved in important biological functions, mainly related to neurodevelopment [98]. Western diets associated with obesity are characterized by a ratio from 15/1 to 20/1 (n-6/n-3), although an optimal ratio of 4/1 or 1/1 is considered as desirable, given the competence of n-3 and n-6 LCPUFAs biosynthetic pathways [99]. ALA is found in many plant products, such as walnuts, flaxseeds, chia seeds, soybean oil and canola oil. Conversion of ALA to n-3 LCPUFAs (EPA and DHA) is poor (<15%; primarily occurring in the liver) and dependent on the intake of n-6/n-3, gender, and genetic polymorphisms in desaturases/elongases [100,101]. Therefore, direct consumption of EPA and DHA (the main metabolic effectors of n-3 fatty acid essentiality) is obtained from eating cold-water oily fish or from supplements such as fish oil. Additionally, fish oil supplements containing DHA+EPA at variable doses are also used. It is worth mentioning that side effects of high doses n-3 supplements are usually mild and are related to antiplatelet effects [102].

Adequate intakes (AIs) are reported only to ALA because it is the only omega-3 that is considered as essential. According to the 2015–2020 Dietary Guidelines for Americans [51], ALA adequate intake in children ranges from 0.7 g/day in 1–3 years old (both male and female), 0.9 g/day in 4–8 years old (both male and female), 1 g/day (9–13 years old; female) and 1.2 g/day (9–13 years old; male). From 14 years old and adulthood, the AIs for ALA are 1.1 g/day (female) and 1.6 g/day (male) [51]. The European Food Safety Authority (EFSA) established AIs for ALA of 0.5% of energy intake [103]. For DHA + EPA, EFSA set AIs of 100 mg/day for the first year of life and 250 mg/day from the age of 2 years and adulthood (many of the RCTs mentioned in the next paragraph and Table 1 are above these limits). There are reports recommending around 0.25–2 g/day in adults, which corresponds to the amount achieved with two portions/week of fish, with at least one being a portion of fatty fish [101].

Intake of Omega-3 LCPUFAs EPA/DHA may contribute to prevent/manage NAFLD through several mechanisms involving induction of fatty acid oxidation and reduction of de novo lipogenesis (acting as endogenous ligands of peroxisome proliferator-activated receptor-α PPARα; which is also activated by fibrates), and simultaneous inhibition of the transcriptional regulators Carbohydrate Response-Element Binding protein (ChREBP) and Sterol Response Element Binding Protein 1c (SREBP1c) [98]. Omega-3 LCPUFAs also reduce inflammation as they are precursors of mildly inflammatory or anti-inflammatory eicosanoids, coupled to an inhibitory effect on the production of AA-derived inflammatory eicosanoids [98]. Additionally, n-3 LCPUFAs may also protect against NAFLD through the modulation of microbiota and the reduction of endotoxemia [101]. On the other hand, low consumption of n-3 LCPUFAs and/or high n-6/n-3 ratios are risk factors for NAFLD development [104]. In children, a cross-sectional study found that most children with NAFLD have insufficient intake of n-3 LCPUFAs (<200 mg/day) [105]. Several studies have tested the beneficial effects of n-3 LCPUFAs administration on NAFLD, both in adults and children/adolescents [91,101,106]. A randomized controlled trial using 250 or 500 mg of DHA versus placebo study conducted in 60 children with NAFLD for two years indicated that DHA administration was effective in reducing fatty liver steatosis, possibly exerting anti-inflammatory actions via the G protein-coupled receptor GPR120 [107,108,109]. Boyraz et al. [110] also found a reduction in hepatic steatosis and improvement of plasma liver enzymes using 1 g/day of n-3 LCPUFAs during a year. Positive results were also found by Pacifico et al. [111] using (250 mg oil/day, 39% DHA) vs. placebo with decreased liver and visceral fat, improvements in plasma triglycerides and insulin sensitivity, but without changes in BMI and liver enzymes. In contrast, a randomized intervention using 450–1300 mg/day of EPA + DHA for six months in children with NAFLD did not reduce the Alanine aminotransferase (ALT) liver enzyme levels (primary aim of the study), although a reduction in the aspartate aminotransferase (AST) liver enzyme level was achieved [112]. In this study, no differences were found in liver hyperechogenicity, insulin resistance, or serum lipid levels. It was proposed that the conduction of larger trials in children with NAFLD involving longer periods and only focused on DHA are necessary. A meta-analysis of randomized controlled trials in children concluded that the administration of n-3 LCPUFA might reduce liver steatosis and liver function markers, representing a possible nutritional approach to treat NAFLD in children. More recently, Spahis et al. [113] compared children with NAFLD with different degrees of severity in response to n-3 LCPUFA supplementation (2 g/day for 1 year) finding important reductions of the hepatic steatosis, fatty liver index, hepatic enzyme activities (ALT and ALT/AST ratio), lipid profile, oxidative markers and carotid intima media thickness, while enhancing circulating adiponectin in the n-3 LCPUFA group compared to the placebo. Another randomized trial found that the combination of DHA, choline and vitamin E may reduce steatosis, glycemia and liver enzyme activities in children with NASH [114]. The most recent meta-analyses of studies in adults and children reaffirm the positive effects of n-3 LCPUFAs on NAFLD, and proposed long-term studies focusing on NASH [115,116,117,118]. The use of different doses of EPA and DHA, low conversion of DHA from EPA and genetic polymorphisms may also play a role in explaining differences in intervention trials using n-3 LCPUFAs [115]. A recent review article concluded that n-3 PUFAs supplementation is a safe, viable and effective intervention that reduces intrahepatic fat content in both children and adults [115].

### 2.3. Vitamin E

Originally, the pathogenesis of NAFLD was described by the two-hit hypothesis, which argues that insulin resistance leads to hepatic steatosis and oxidative stress leads to steatohepatitis and fibrosis [119]. The crucial role of oxidative stress in the progression of NAFLD to more advanced stages is related to the production of reactive oxygen species, that it is a consequence of excess fatty acids in hepatic cells with energy depletion and the subsequent mitochondrial dysfunction [120,121]. Later, this theory was expanded into the multi-hits hypothesis, suggesting that in NAFLD pathogenesis, many hits may act in parallel, especially factors derived from gut and adipose tissue, resulting in endoplasmic stress, oxidative stress, and hepatocyte apoptosis [122]. NAFLD affects the antilipolytic action of insulin and promotes the production of excess free fatty acids (FFAs), increasing the delivery of FFAs to the liver and de novo lipogenesis, and developing secondary insulin resistance [123]. A study published in 2006 demonstrated lower serum vitamin C and alpha-tocopherol concentrations and higher lipid peroxides in patients with NAFLD and metabolic syndrome compared to controls [124].

Since oxidative stress is one of the initiating factors of lipid peroxidation and subsequent hepatocellular damage in NAFLD, antioxidants such as vitamin E might have beneficial effects against the disease. Vitamin E is a broad term that includes four tocopherols (α-, β-, γ-, and δ-tocopherols) and four tocotrienols (α-, β-, γ-, and δ-tocotrienols) that are present in different nutrients [125]. The major dietary sources of vitamin E are vegetable oils, being safflower and sunflower oils high in α-tocopherol. Vitamin E is considered a major lipid- soluble chain-breaking antioxidant that may act as a scavenger of hydroxyl, peroxyl, and superoxide radicals and terminate the oxidation of polyunsaturated fatty acids, protecting against plasma lipid and low-density lipoprotein peroxidation. Its biological action involves stopping the chain reaction of peroxyl radical production and preventing the further oxidation of PUFAs in the membrane. In addition to its strong antioxidant effects, Vitamin E is a potential alternative for the treatment of NAFLD and NASH for its non-antioxidant effects. In diet-induced NASH, it is demonstrated that Vitamin E can reduce inflammation, improve hepatic fibrosis, and attenuate hyperinsulinemia, without affecting the insulin signaling [126]. Other mechanisms of action include induction of adiponectin expression, reduction of the inflammatory pathway, and regulation of macrophage polarization [126,127].

In a prospective, cross-sectional registry-based study [128], Vos et al. demonstrated that children with NAFLD have a diet that is low in vitamin E, which may contribute to the pathophysiology of the disease. Furthermore, different studies have evaluated the efficacy of Vitamin E in the management of NAFLD; in 2004, Vajro et al. [129] compared the effect of vitamin E (400 mg/day × 2 months, 100 mg/day × 3 months) on transaminases values and ultrasonographic bright liver in a controlled group of children with suspected NAFLD, showing no benefits in the use of the antioxidant therapy. The Italian group of Nobili et al. developed a study [130] comparing the effect of a nutritional program alone or combined with alpha-tocopherol (600 IU/day) and ascorbic acid (500 mg/day) on liver enzymes and insulin resistance in biopsy-diagnosis NAFLD children (*n* = 90). After 12 months, they found that diet and physical activity seem to lead to a significant improvement of liver function beyond antioxidant treatment. In a extension study [35], the authors evaluated in 53 patients (age 5.7–18.8 years) the effect of lifestyle intervention plus alpha-tocopherol and ascorbic acid (same doses that in the original study) (*n* = 25) or placebo (*n* = 28) for 24 months. At the end of the study, they found that lifestyle intervention was associated with a significant improvement in liver histology and laboratory abnormalities, with no differences in the group that received alpha-tocopherol plus ascorbic acid. More recently, in a double blind, placebo-controlled clinical trial that aimed to evaluate the impact of vitamin E and metformin in children and adolescents with NAFLD diagnosed by biopsy [131], the authors included 173 patients (aged 8–17 years) who received a daily dosing of 800 IU of vitamin E (*n* = 58), 1000 mg of metformin (*n* = 57) or a placebo (*n* = 58) for 96 weeks. They found no significant improvement in ALT although there was an improvement in NAFLD activity score and hepatocellular ballooning score in the liver biopsies. Cho et al. [132] determined that for children with NAFLD, the reduction of the BMI through diet and exercise plus the use of vitamin E and ursodeoxycholic acid has greater effect on the improvement of hepatic biochemical profile (AST, ALT, AST/ALT ratio, alkaline phosphatase, total bilirubin and γ-glutamyl transpeptidase) when compared to BMI reduction alone. Another study [133] aiming to evaluate the effect of antioxidant supplementation on biomarkers of oxidative stress, inflammation, and liver function, randomly assigned 44 overweight/obese children aged 10–17 years to a intervention with daily antioxidants (vitamin E, 400 IU; vitamin C, 500 mg; selenium, 50 mg) or placebo for four months. At the end of the study, it was observed that there was a significant effect of antioxidant supplementation on antioxidant status and oxidative stress and also the liver transaminases were moderately improved in the treatment group in comparison with the controls. Similarly, Wang et al. [134] studied the effects of vitamin E and lifestyle on ALT levels in 76 Chinese children (ages 10–17 years) with obesity and NAFLD, finding that vitamin E was effective in reducing ALT; however, lifestyle intervention was more effective. A systematic review and meta-analyses of randomized control trials conducted in adults and children published in 2019 [135] that included four of the studies mentioned above [35,129,130,131], concludes that vitamin E therapy provides significant biochemical and histological improvements in adult patients with NAFLD, while in pediatric patients its use showed insignificant efficacy compared with placebo.

It is important to consider that some evidence exists against the use of Vitamin E; one study has shown that the daily administration of vitamin E increased the risk of developing prostate cancer, and even some studies related high-dose vitamin E supplementation with increased mortality [136,137,138]. The 2017 NASPGHAN Clinical Practice Guideline for the Diagnosis and Treatment of Non-alcoholic Fatty Liver Disease in Children [36] stated no currently available medications or supplements are recommended to treat NAFLD because of a lack of evidence. Despite the 2018 American Association for the Study of Liver Diseases practice guidance supporting the use of vitamin E in children with biopsy-proven NASH [139], the safety and efficacy of vitamin E for the treatment of NAFLD, particularly in the pediatric population, requires further studies before its use can be recommended in clinical practice.

## 3. Conclusions

Non-Alcoholic Fatty Liver Disease is the most common form of chronic liver disease; its pathophysiology is complex and involves a series of different factors that influence its presentation and development. The evidence shows that restriction of carbohydrates, fructose, free sugars or SSB are adequate interventions to prevent NAFLD in the pediatric population, mainly as a strategy to decrease adiposity. Additionally, the use of omega-3 long-chain fatty acids (achieving DHA + EPA intake of 250 mg/day) is a safe intervention to improve the clinical course of children with NAFLD, whereas the use of antioxidants like Vitamin E require more studies to be universally recommended. Until there is a better quality of evidence, the management of pediatric NAFLD should be focused on lifestyle modification.

## Figures and Tables

**Table 1:** Summary of the published randomized clinical trials and interventions that evaluate the effect of reduction in fructose consumption, omega 3 fatty acids supplementation and Vitamin E supplementation in children with NAFLD.

Reduction in Fructose Consumption				
Author, Year, Country	Sample Size and Age Range	Intervention and Duration	Outcomes	Results
Jin et al. [88], 2014, USA	24 overweight adolescents (11–18 years) with hepatic fat >8% on MRS	Calorie-matched study-provided fructose only or glucose only beverages (3 × 8 fl bottles per day), for 4 weeks	(1) Hepatic steatosis (measured by MRS), ALT, AST (2) Associated cardiovascular risk factors (BW, TG, FI, HOMA-IR, FFA, LDL)	No significant change in hepatic fat, liver enzymes or body weight in either group. In the glucose beverage group there was significantly improved HOMA-IR, hs-CRP, and LDL oxidation.
Schwarz et al. [87], 2017, USA	Obese Children (9–18 years old; *n* = 41) with habitual high sugar consumption	9 days of isocaloric fructose restriction	(1) Liver, visceral and subcutaneous fat (2) de novo lipogenesis (DNL)	Reductions in liver fat, visceral fat and DNL after fructose restriction
Jin et al. [96], 2012, USA	Nine children with NAFLD and 10 matched controls without NAFLD (11–18 years old)	24-h periods of isocaloric macronutrient-balanced meals with either a fructose beverage or a glucose beverage	Plasma glucose, insulin, triglyceride, apolipoprotein B, high-density lipoprotein cholesterol, and nonesterified free fatty acid levels.	Effects of fructose on plasma lipids occurred in both healthy and NAFLD children, being the latter more sensitive to fructose actions
Jin et al. [95], 2014, USA	As in Jin et al. [88,96]	As in Jin et al. [88,96]	As in Jin et al. [88,96]	Endotoxin levels in NAFLD increased after fructose beverages compared to healthy children.
**Omega 3 Fatty Acids**				
Nobili et al. [107,108], 2011, 2013, Italy	60, <18 years	DHA 500 mg/day, or DHA 250 mg/day, or placebo for 24 months (with evaluations at 6, 12, 18, and 24 months)	(1) Change in liver fat (detected by ultrasonography) (2) TG, ALT, BMI, HOMA-IR	The severity of liver steatosis decreased in both treated groups vs. placebo (OR ≤ 0.02, *p* ≤ 0.05). TG were lower in both the treated groups. ALT was lower in the treatment groups from month 12 onwards. HOMA was lower in the DHA 250 mg group vs. placebo at 6 and 12 months.
Boyraz et al. [110], 2015, Turkey	108 obese adolescents, 9–17 years	1000 mg/day omega-3 plus diet (50% carbohydrates, 20% protein and 30% fat) plus lifestyle intervention (exercise three times per week for 1 h and the promotion of self-initiated physical activities) or placebo plus diet plus lifestyle intervention for 12 months	(1) US, ALT, AST (2) BMI, HDL-C, TG, TC, FI, FG, IS, HOMA-IR, SBP	Improvement of steatosis, ALT and AST in both groups. Increase HDL-C, improvement of TG, FI and HOMA-IR. Improvement in BMI in the intervention and control groups. Improvement of SBP in the intervention group.
Janczyk et al. [112], 2015, Poland	76	450–1300 mg/day 3:2 DHA:EPA plus individually prescribed diet and PA versus omega 6 sunflower oil for 6 months	(1) ALT, AST, GGT (2) FSG, FI, HOMA- IR, adiponectin, cholesterol, steatosis (US), BMI, WC	Lower ALT levels in both groups the intervention and control (but without statistical difference between the groups). Lower AST and GGT levels in the intervention group. No changes in ALT, steatosis, FSG, FI, HOMA-IR, BMI z score or WC z score in both groups.
Pacifico et al. [111], 2015, Italy	51, <18 years	250 mg/day DHA plus low-calorie diet plus 60 min PA 5 times a week versus placebo (290 mg/day linoleic acid) for 6 months	(1) Hepatic fat, ALT (2) TG, Z-BMI, FI, HOMA-IR	Lower hepatic fat by MRS, FI, and TG in the intervention group.
**Vitamin E**				
Vajro et al. [129], 2004, Italy	28 obese participants, age range NR	Oral a-acetatetocopherol (400 mg/day for 2 months, 100 mg/day for 3 months) plus low-calorie diet plus exercise versus placebo	(1) Liver echogenicity, ALT (2) BMI	Results were stratified based on weight loss: decrease BMI plus vitamin E: improved liver echogenicity, and ALT levels. Stable BMI plus vitamin E: improved the liver echogenicity.
Nobili et al. [35,130], 2006, 2008, Italy	90 participants, 3–18 years	Alpha-tocopherol (600 ID/d) plus ascorbic acid (500 mg/day) plus hypo-/isocaloric diet plus 45 min/day aerobic exercise versus placebo for 24 months	(1) NAFLD Activity Score, ALT, AST (2) Z-BMI, BMI, TG, TC, FSG, FI, HOMA-IR	Improvement in NAFLD activity score, ALT, and AST in both the intervention and control groups (but without statistical difference between the groups).
Wang et al. [134], 2008, China	76 obese children with NAFLD, 10–17 years	Three groups: Vitamin E (100 mg/day) versus life-style interventions (low calorie diet + exercise + reduced caloric intake) versus no intervention	(1) US, ALT, AST (2) Z-BMI, TG, TC, FSG, FI, HOMA	No change to liver echogenicity in any group. Reduction in ALT, AST, BMI, TG, TC, FI, FSG and HOMA- IR in the intervention group.
Lavine et al. [131], 2011, USA	173 participants, 8–17 years	800 IU of vitamin E (58 patients), 1000 mg of metformin (57 patients), or placebo (58 patients) for 96 weeks	(1) ALT (2) NAFLD activity score, QOL, Z-BMI, BMI, BW, WC, TG, TC, FSG, HOMA-IR, HDL, LDL	No significant improvement in ALT levels between the groups. Improved the NAFLD activity score in the intervention group. Resolution of NASH was significantly greater in the children treated with vitamin E versus the placebo group.
Murer et al. [133], 2014, Switzerland	44 overweight or obese children and adolescents, 10–18 years	Daily antioxidants (vitamin E, 400 IU; vitamin C, 500 mg; selenium, 50 μg) plus lifestyle modifications or placebo for 4 months	(1) Biomarkers of oxidative stress, inflammation, and liver function	Significant treatment effect of antioxidant supplementation on antioxidant status and on ALT.

ALT: Alanine aminotransferase; AST: Aspartate aminotransferase; BMI: body mass index; DHA: docosahexanoic acid; EPA: eicosapentaenoic; FI: fasting insulin; FSG: fasting serum glucose; GGT: gamma-glutamyl transpeptidase; HDL: high-density lipoprotein; HOMA-IR: homeostatic model assessment of insulin resistance; IU: international units; LDL: low-density lipoprotein; MRS: magnetic resonance spectroscopic imaging; NR: not reported; OR: odds ratio; PA: physical activity; QOL: quality of life; SBP: systolic blood pressure; TC: total cholesterol; TG: triglycerides; US: ultrasound; VAT: visceral adipose tissue; WC: waist circumference; Z-BMI: BMI z score.

## References

[1] Eslam M., Newsome P.N., Sarin S.K., Anstee Q.M., Targher G., Romero-Gomez M., Zelber-Sagi S., Wai-Sun Wong V., Dufour J.-F., Schattenberg J.M. (2020). A new definition for metabolic dysfunction-associated fatty liver disease: An international expert consensus statement. J. Hepatol..

[2] Younossi Z.M. (2019). Non-alcoholic fatty liver disease—A global public health perspective. J. Hepatol..

[3] Sanyal A.J., Harrison S.A., Ratziu V., Abdelmalek M.F., Diehl A.M., Caldwell S., Shiffman M.L., Schall R.A., Jia C., McColgan B. (2019). The Natural History of Advanced Fibrosis due to Nonalcoholic Steatohepatitis: Data from the Simtuzumab Trials. Hepatology.

[4] Kovalic A.J., Banerjee P., Tran Q.T., Singal A.K., Satapathy S.K. (2018). Genetic and Epigenetic Culprits in the Pathogenesis of Nonalcoholic Fatty Liver Disease. J. Clin. Exp. Hepatol..

[5] Cotter T.G., Rinella M. (2020). Nonalcoholic Fatty Liver Disease 2020: The State of the Disease. Gastroenterology.

[6] Rinella M.E. (2015). Nonalcoholic fatty liver disease: A systematic review. JAMA.

[7] Tricò D., Caprio S., Rosaria Umano G., Pierpont B., Nouws J., Galderisi A., Kim G., Mata M.M., Santoro N. (2018). Metabolic Features of Nonalcoholic Fatty Liver (NAFL) in Obese Adolescents: Findings from a Multiethnic Cohort. Hepatology.

[8] Anderson E.L., Howe L.D., Jones H.E., Higgins J.P.T., Lawlor D.A., Fraser A. (2015). The Prevalence of Non-Alcoholic Fatty Liver Disease in Children and Adolescents: A Systematic Review and Meta-Analysis. PLoS ONE.

[9] Schwimmer J.B., Deutsch R., Kahen T., Lavine J.E., Stanley C., Behling C. (2006). Prevalence of fatty liver in children and adolescents. Pediatrics.

[10] Braun H.A., Faasse S.A., Vos M.B. (2018). Advances in Pediatric Fatty Liver Disease: Pathogenesis, Diagnosis, and Treatment. Gastroenterol. Clin. N. Am..

[11] Kotronen A., Yki-Järvinen H. (2008). Fatty liver: A novel component of the metabolic syndrome. Arterioscler. Thromb. Vasc. Biol..

[12] Marchesini G., Bugianesi E., Forlani G., Cerrelli F., Lenzi M., Manini R., Natale S., Vanni E., Villanova N., Melchionda N. (2003). Nonalcoholic fatty liver, steatohepatitis, and the metabolic syndrome. Hepatology.

[13] Manco M., Bedogni G., Marcellini M., Devito R., Ciampalini P., Sartorelli M.R., Comparcola D., Piemonte F., Nobili V. (2008). Waist circumference correlates with liver fibrosis in children with non-alcoholic steatohepatitis. Gut.

[14] Lee S., Rivera-Vega M., Alsayed H.M.A.A., Boesch C., Libman I. (2015). Metabolic inflexibility and insulin resistance in obese adolescents with non-alcoholic fatty liver disease. Pediatr. Diabetes.

[15] Schwimmer J.B., Deutsch R., Rauch J.B., Behling C., Newbury R., Lavine J.E. (2003). Obesity, insulin resistance, and other clinicopathological correlates of pediatric nonalcoholic fatty liver disease. J. Pediatr..

[16] Dharmalingam M., Yamasandhi P. (2018). Nonalcoholic fatty liver disease and Type 2 diabetes mellitus. Indian J. Endocrinol. Metab..

[17] Hazlehurst J.M., Woods C., Marjot T., Cobbold J.F., Tomlinson J.W. (2016). Non-alcoholic fatty liver disease and diabetes. Metabolism.

[18] Mantovani A., Byrne C.D., Bonora E., Targher G. (2018). Nonalcoholic fatty liver disease and risk of incident type 2 diabetes: A meta-analysis. Diabetes Care.

[19] Sao R., Aronow W.S. (2018). Association of non-alcoholic fatty liver disease with cardiovascular disease and subclinical atherosclerosis. Arch. Med. Sci..

[20] Madan S.A., John F., Pyrsopoulos N., Pitchumoni C.S. (2015). Nonalcoholic fatty liver disease and carotid artery atherosclerosis in children and adults: A meta-analysis. Eur. J. Gastroenterol. Hepatol..

[21] Targher G., Day C.P., Bonora E. (2010). Risk of cardiovascular disease in patients with nonalcoholic fatty liver disease. N. Engl. J. Med..

[22] Feldstein A.E., Charatcharoenwitthaya P., Treeprasertsuk S., Benson J.T., Enders F.B., Angulo P. (2009). The natural history of non-alcoholic fatty liver disease in children: A follow-up study for up to 20 years. Gut.

[23] Dulai P.S., Singh S., Patel J., Soni M., Prokop L.J., Younossi Z., Sebastiani G., Ekstedt M., Hagstrom H., Nasr P. (2017). Increased risk of mortality by fibrosis stage in nonalcoholic fatty liver disease: Systematic review and meta-analysis. Hepatology.

[24] Younossi Z.M., Marchesini G., Pinto-Cortez H., Petta S. (2019). Epidemiology of Nonalcoholic Fatty Liver Disease and Nonalcoholic Steatohepatitis: Implications for Liver Transplantation. Transplantation.

[25] Noureddin M., Vipani A., Bresee C., Todo T., Kim I.K., Alkhouri N., Setiawan V.W., Tran T., Ayoub W.S., Lu S.C. (2018). NASH Leading Cause of Liver Transplant in Women: Updated Analysis of Indications for Liver Transplant and Ethnic and Gender Variances. Am. J. Gastroenterol..

[26] Benítez C., Wolff R. (2018). Current Status and Future Challenges of Liver Transplantation Programs in Chile. Liver Transpl..

[27] Wong R.J., Cheung R., Ahmed A. (2014). Nonalcoholic steatohepatitis is the most rapidly growing indication for liver transplantation in patients with hepatocellular carcinoma in the U.S. Hepatology.

[28] Sayiner M., Otgonsuren M., Cable R., Younossi I., Afendy M., Golabi P., Henry L., Younossi Z.M. (2017). Variables Associated with Inpatient and Outpatient Resource Utilization among Medicare Beneficiaries with Nonalcoholic Fatty Liver Disease with or without Cirrhosis. J. Clin. Gastroenterol..

[29] Younossi Z.M., Zheng L., Stepanova M., Venkatesan C., Mishra A. (2014). Clinical outcomes and resource utilisation in Medicare patients with chronic liver disease: A historical cohort study. BMJ Open.

[30] Younossi Z.M. (2018). Patient-Reported Outcomes and the Economic Effects of Nonalcoholic Fatty Liver Disease and Nonalcoholic Steatohepatitis: The Value Proposition. Hepatology.

[31] European Association for the Study of the Liver (EASL), European Association for the Study of Diabetes (EASD) (2016). European Association for the Study of Obesity (EASO) EASL–EASD–EASO Clinical Practice Guidelines for the management of non-alcoholic fatty liver disease. J. Hepatol..

[32] Younossi Z.M., Henry L., Bush H., Mishra A. (2018). Clinical and Economic Burden of Nonalcoholic Fatty Liver Disease and Nonalcoholic Steatohepatitis. Clin. Liver Dis..

[33] Shetty A., Syn W.-K. (2019). Current treatment options for nonalcoholic fatty liver disease. Curr. Opin. Gastroenterol..

[34] Vilar-Gomez E., Martinez-Perez Y., Calzadilla-Bertot L., Torres-Gonzalez A., Gra-Oramas B., Gonzalez-Fabian L., Friedman S.L., Diago M., Romero-Gomez M. (2015). Weight loss through lifestyle modification significantly reduces features of nonalcoholic steatohepatitis. Gastroenterology.

[35] Nobili V., Manco M., Devito R., Di Ciommo V., Comparcola D., Sartorelli M.R., Piemonte F., Marcellini M., Angulo P. (2008). Lifestyle intervention and antioxidant therapy in children with nonalcoholic fatty liver disease: A randomized, controlled trial. Hepatology.

[36] Vos M.B., Abrams S.H., Barlow S.E., Caprio S., Daniels S.R., Kohli R., Mouzaki M., Sathya P., Schwimmer J.B., Sundaram S.S. (2017). NASPGHAN Clinical Practice Guideline for the Diagnosis and Treatment of Nonalcoholic Fatty Liver Disease in Children: Recommendations from the Expert Committee on NAFLD (ECON) and the North American Society of Pediatric Gastroenterology, Hepatology and Nu. J. Pediatr. Gastroenterol. Nutr..

[37] Perumpail B., Li A., John N., Sallam S., Shah N., Kwong W., Cholankeril G., Kim D., Ahmed A. (2019). The Therapeutic Implications of the Gut Microbiome and Probiotics in Patients with NAFLD. Diseases.

[38] Zhou R., Fan X., Schnabl B. (2019). Role of the intestinal microbiome in liver fibrosis development and new treatment strategies. Transl. Res..

[39] Yan J.-H., Guan B.-J., Gao H.-Y., Peng X.-E. (2018). Omega-3 polyunsaturated fatty acid supplementation and non-alcoholic fatty liver disease: A meta-analysis of randomized controlled trials. Medicine.

[40] Mann J.P., Tang G.Y., Nobili V., Armstrong M.J. (2018). Evaluations of Lifestyle, Dietary, and Pharmacologic Treatments for Pediatric Nonalcoholic Fatty Liver Disease: A Systematic Review. Clin. Gastroenterol. Hepatol..

[41] AlKhater S.A. (2015). Paediatric non-alcoholic fatty liver disease: An overview. Obes. Rev..

[42] Nobili V., Alkhouri N., Alisi A., Della Corte C., Fitzpatrick E., Raponi M., Dhawan A. (2015). Nonalcoholic fatty liver disease: A challenge for pediatricians. JAMA Pediatr..

[43] Ogden C.L., Fryar C.D., Hales C.M., Carroll M.D., Aoki Y., Freedman D.S. (2018). Differences in obesity Prevalence by Demographics and Urbanization in US Children and Adolescents, 2013–2016. JAMA.

[44] Fidler Mis N., Braegger C., Bronsky J., Campoy C., Domellöf M., Embleton N.D., Hojsak I., Hulst J., Indrio F., Lapillonne A. (2017). Sugar in Infants, Children and Adolescents: A Position Paper of the European Society for Paediatric Gastroenterology, Hepatology and Nutrition Committee on Nutrition. J. Pediatr. Gastroenterol. Nutr..

[45] Vos M.B., Kaar J.L., Welsh J.A., Van Horn L.V., Feig D.I., Anderson C.A.M., Patel M.J., Cruz Munos J., Krebs N.F., Xanthakos S.A. (2017). Added sugars and cardiovascular disease risk in children: A scientific statement from the American Heart Association. Circulation.

[46] Popkin B.M., Hawkes C. (2016). Sweetening of the global diet, particularly beverages: Patterns, trends, and policy responses. Lancet Diabetes Endocrinol..

[47] Luo S., Monterosso J.R., Sarpelleh K., Page K.A. (2015). Differential effects of fructose versus glucose on brain and appetitive responses to food cues and decisions for food rewards. Proc. Natl. Acad. Sci. USA.

[48] Pan A., Hu F.B. (2011). Effects of carbohydrates on satiety: Differences between liquid and solid food. Curr. Opin. Clin. Nutr. Metab. Care.

[49] Sylvetsky A.C., Visek A.J., Halberg S., Rhee D.K., Ongaro Z., Essel K.D., Dietz W.H., Sacheck J. (2020). Beyond taste and easy access: Physical, cognitive, interpersonal, and emotional reasons for sugary drink consumption among children and adolescents. Appetite.

[50] (2015). World Health Organization Guideline: Sugars Intake for Adults and Children.

[51] U.S. Department of Health and Human Services, U.S. Department of Agriculture (2015). 2015–2020 Dietary Guidelines for Americans. 8th Edition. http://health.gov/dietaryguidelines/2015/guidelines/.

[52] Vitolo M.R. (2018). How Much Free Sugars Intake Should Be Recommended for Children Younger Than 2 Years Old?. J. Pediatr. Gastroenterol. Nutr..

[53] Tondt J., Yancy W.S., Westman E.C. (2020). Application of Nutrient Essentiality Criteria to Dietary Carbohydrates. Nutr. Res. Rev..

[54] Hannou S.A., Haslam D.E., McKeown N.M., Herman M.A. (2018). Fructose metabolism and metabolic disease. J. Clin. Investig..

[55] Lustig R.H. (2010). Fructose: Metabolic, hedonic, and societal parallels with ethanol. J. Am. Diet. Assoc..

[56] Taskinen M.R., Packard C.J., Borén J. (2019). Dietary fructose and the metabolic syndrome. Nutrients.

[57] Jang C., Hui S., Lu W., Cowan A.J., Morscher R.J., Lee G., Liu W., Tesz G.J., Birnbaum M.J., Rabinowitz J.D. (2018). The Small Intestine Converts Dietary Fructose into Glucose and Organic Acids. Cell Metab..

[58] Gonzalez J.T., Betts J.A. (2018). Dietary Fructose Metabolism by Splanchnic Organs: Size Matters. Cell Metab..

[59] Chong M.F.F., Fielding B.A., Frayn K.N. (2007). Mechanisms for the acute effect of fructose on postprandial lipemia. Am. J. Clin. Nutr..

[60] Sun S.Z., Empie M.W. (2012). Fructose metabolism in humans—What isotopic tracer studies tell us. Nutr. Metab..

[61] Chiu S., Mulligan K., Schwarz J.M. (2018). Dietary carbohydrates and fatty liver disease: De novo lipogenesis. Curr. Opin. Clin. Nutr. Metab. Care.

[62] Stanhope K.L., Schwarz J.M., Keim N.L., Griffen S.C., Bremer A.A., Graham J.L., Hatcher B., Cox C.L., Dyachenko A., Zhang W. (2009). Consuming fructose-sweetened, not glucose-sweetened, beverages increases visceral adiposity and lipids and decreases insulin sensitivity in overweight/obese humans. J. Clin. Investig..

[63] Johnson R.J., Nakagawa T., Sanchez-Lozada L.G., Shafiu M., Sundaram S., Le M., Ishimoto T., Sautin Y.Y., Lanaspa M.A. (2013). Sugar, uric acid, and the etiology of diabetes and obesity. Diabetes.

[64] Dushay J.R., Toschi E., Mitten E.K., Fisher F.M., Herman M.A., Maratos-Flier E. (2015). Fructose ingestion acutely stimulates circulating FGF21 levels in humans. Mol. Metab..

[65] Taskinen M.R., Söderlund S., Bogl L.H., Hakkarainen A., Matikainen N., Pietiläinen K.H., Räsänen S., Lundbom N., Björnson E., Eliasson B. (2017). Adverse effects of fructose on cardiometabolic risk factors and hepatic lipid metabolism in subjects with abdominal obesity. J. Intern. Med..

[66] Cortese A., Zhu Y., Rebelo A.P., Negri S., Courel S., Abreu L., Bacon C.J., Bai Y., Bis-Brewer D.M., Bugiardini E. (2020). Biallelic mutations in SORD cause a common and potentially treatable hereditary neuropathy with implications for diabetes. Nat. Genet..

[67] Sanchez-Lozada L.G., Andres-Hernando A., Garcia-Arroyo F.E., Cicerchi C., Li N., Kuwabara M., Roncal-Jimenez C.A., Johnson R.J., Lanaspa M.A. (2019). Uric acid activates aldose reductase and the polyol pathway for endogenous fructose and fat production causing development of fatty liver in rats. J. Biol. Chem..

[68] Russo E., Leoncini G., Esposito P., Garibotto G., Pontremoli R., Viazzi F. (2020). Fructose and uric acid: Major mediators of cardiovascular disease risk starting at pediatric age. Int. J. Mol. Sci..

[69] Nier A., Brandt A., Conzelmann I.B., Özel Y., Bergheim I. (2018). Non-alcoholic fatty liver disease in overweight children: Role of fructose intake and dietary pattern. Nutrients.

[70] Mosca A., Nobili V., De Vito R., Crudele A., Scorletti E., Villani A., Alisi A., Byrne C.D. (2017). Serum uric acid concentrations and fructose consumption are independently associated with NASH in children and adolescents. J. Hepatol..

[71] Qiu L., Guo C. (2019). Natural Aldose Reductase Inhibitor: A Potential Therapeutic Agent for Non-Alcoholic Fatty Liver Disease. Curr. Drug Targets.

[72] Lustig R.H., Schmidt L.A., Brindis C.D. (2012). Public health: The toxic truth about sugar. Nature.

[73] Sievenpiper J.L., Tappy L., Brouns F. (2015). Fructose as a Driver of Diabetes: An Incomplete View of the Evidence. Mayo Clin. Proc..

[74] Malik V.S., Hu F.B. (2015). Fructose and cardiometabolic Health What the Evidence from Sugar-Sweetened Beverages Tells Us. J. Am. Coll. Cardiol..

[75] Evans R.A., Frese M., Romero J., Cunningham J.H., Mills K.E. (2017). Chronic fructose substitution for glucose or sucrose in food or beverages has little effect on fasting blood glucose, insulin, or triglycerides: A systematic review and meta-analysis. Am. J. Clin. Nutr..

[76] Evans R.A., Frese M., Romero J., Cunningham J.H., Mills K.E. (2017). Fructose replacement of glucose or sucrose in food or beverages lowers postprandial glucose and insulin without raising triglycerides: A systematic review and meta-analysis. Am. J. Clin. Nutr..

[77] Sievenpiper J.L. (2017). Fructose: Back to the future?. Am. J. Clin. Nutr..

[78] Semnani-Azad Z., Khan T.A., Blanco Mejia S., De Souza R.J., Leiter L.A., Kendall C.W.C., Hanley A.J., Sievenpiper J.L. (2020). Association of Major Food Sources of Fructose-Containing Sugars with Incident Metabolic Syndrome: A Systematic Review and Meta-Analysis. JAMA Netw. Open.

[79] Ebbeling C.B., Feldman H.A., Chomitz V.R., Antonelli T.A., Gortmaker S.L., Osganian S.K., Ludwig D.S. (2012). A randomized trial of sugar-sweetened beverages and adolescent body weight. N. Engl. J. Med..

[80] De Ruyter J.C., Olthof M.R., Seidell J.C., Katan M.B. (2012). A trial of sugar-free or sugar-sweetened beverages and body weight in children. N. Engl. J. Med..

[81] Wehmeyer M.H., Zyriax B.C., Jagemann B., Roth E., Windler E., Zur Wiesch J.S., Lohse A.W., Kluwe J. (2016). Nonalcoholic fatty liver disease is associated with excessive calorie intake rather than a distinctive dietary pattern. Medicine.

[82] Jin R., Vos M.B. (2015). Fructose and liver function—Is this behind nonalcoholic liver disease?. Curr. Opin. Clin. Nutr. Metab. Care.

[83] Jegatheesan P., De Bandt J.P. (2017). Fructose and NAFLD: The multifaceted aspects of fructose metabolism. Nutrients.

[84] Lim J.S., Mietus-Snyder M., Valente A., Schwarz J.M., Lustig R.H. (2010). The role of fructose in the pathogenesis of NAFLD and the metabolic syndrome. Nat. Rev. Gastroenterol. Hepatol..

[85] Jensen T., Abdelmalek M.F., Sullivan S., Nadeau K.J., Green M., Roncal C., Nakagawa T., Kuwabara M., Sato Y., Kang D.H. (2018). Fructose and sugar: A major mediator of non-alcoholic fatty liver disease. J. Hepatol..

[86] Perito E.R., Rodriguez L.A., Lustig R.H. (2013). Dietary treatment of nonalcoholic steatohepatitis. Curr. Opin. Gastroenterol..

[87] Schwarz J.M., Noworolski S.M., Erkin-Cakmak A., Korn N.J., Wen M.J., Tai V.W., Jones G.M., Palii S.P., Velasco-Alin M., Pan K. (2017). Effects of Dietary Fructose Restriction on Liver Fat, De Novo Lipogenesis, and Insulin Kinetics in Children with Obesity. Gastroenterology.

[88] Jin R., Welsh J.A., Le N.-A., Holzberg J., Sharma P., Martin D.R., Vos M.B. (2014). Dietary Fructose Reduction Improves Markers of Cardiovascular Disease Risk in Hispanic-American Adolescents with NAFLD. Nutrients.

[89] Goss A.M., Dowla S., Pendergrass M., Ashraf A., Bolding M., Morrison S., Amerson A., Soleymani T., Gower B. (2020). Effects of a carbohydrate-restricted diet on hepatic lipid content in adolescents with non-alcoholic fatty liver disease: A pilot, randomized trial. Pediatr. Obes..

[90] Mosca A., Della Corte C., Sartorelli M.R., Ferretti F., Nicita F., Vania A., Nobili V. (2016). Beverage consumption and paediatric NAFLD. Eat. Weight Disord..

[91] Panera N., Barbaro B., Della Corte C., Mosca A., Nobili V., Alisi A. (2018). A review of the pathogenic and therapeutic role of nutrition in pediatric nonalcoholic fatty liver disease. Nutr. Res..

[92] Sekkarie A., Welsh J.A., Vos M.B. (2018). Carbohydrates and diet patterns in nonalcoholic fatty liver disease in children and adolescents. Curr. Opin. Clin. Nutr. Metab. Care.

[93] Spruss A., Kanuri G., Wagnerberger S., Haub S., Bischoff S.C., Bergheim I. (2009). Toll-like receptor 4 is involved in the development of fructose-induced hepatic steatosis in mice. Hepatology.

[94] Jiang X., Zheng J., Zhang S., Wang B., Wu C., Guo X. (2020). Advances in the Involvement of Gut Microbiota in Pathophysiology of NAFLD. Front. Med..

[95] Jin R., Willment A., Patel S.S., Sun X., Song M., Mannery Y.O., Kosters A., McClain C.J., Vos M.B. (2014). Fructose Induced Endotoxemia in Pediatric Nonalcoholic Fatty Liver Disease. Int. J. Hepatol..

[96] Jin R., Le N.A., Liu S., Epperson M.F., Ziegler T.R., Welsh J.A., Jones D.P., McClain C.J., Vos M.B. (2012). Children with NAFLD are more sensitive to the adverse metabolic effects of fructose beverages than children without NAFLD. J. Clin. Endocrinol. Metab..

[97] Kakleas K., Christodouli F., Karavanaki K. (2020). Nonalcoholic fatty liver disease, insulin resistance, and sweeteners: A literature review. Expert Rev. Endocrinol. Metab..

[98] Scorletti E., Byrne C.D. (2013). Omega-3 fatty acids, hepatic lipid metabolism, and nonalcoholic fatty liver disease. Annu. Rev. Nutr..

[99] Simopoulos A.P., DiNicolantonio J.J. (2016). The importance of a balanced Ï‰-6 to Ï‰-3 ratio in the prevention and management of obesity. Open Heart.

[100] Glaser C., Heinrich J., Koletzko B. (2010). Role of FADS1 and FADS2 polymorphisms in polyunsaturated fatty acid metabolism. Metabolism.

[101] Scorletti E., Byrne C.D. (2018). Omega-3 fatty acids and non-alcoholic fatty liver disease: Evidence of efficacy and mechanism of action. Mol. Asp. Med..

[102] Lev-Tzion R., Griffiths A.M., Leder O., Turner D. (2014). Omega 3 fatty acids (fish oil) for maintenance of remission in Crohn’s disease. Cochrane Database Syst. Rev..

[103] European Food Safety Authority (EFSA) Dietary Reference Values for the European Union 2019. https://www.efsa.europa.eu/en/topics/topic/dietary-reference-values.

[104] Jump D.B., Depner C.M., Tripathy S., Lytle K.A. (2015). Potential for dietary ω-3 fatty acids to prevent nonalcoholic fatty liver disease and reduce the risk of primary liver cancer. Adv. Nutr..

[105] St-Jules D.E., Watters C.A., Brunt E.M., Wilkens L.R., Novotny R., Belt P., Lavine J.E., Abrams S.H., Himes R., Krisnamurthy R. (2013). Estimation of fish and ω-3 fatty acid intake in pediatric nonalcoholic fatty liver disease. J. Pediatr. Gastroenterol. Nutr..

[106] Corte C.D., Iasevoli S., Strologo A.D., Sanseviero M., Nobili V. (2018). Omega-3 fatty acids and fatty liver disease in children. Advances in Food and Nutrition Research.

[107] Nobili V., Bedogni G., Alisi A., Pietrobattista A., Risé P., Galli C., Agostoni C. (2011). Docosahexaenoic acid supplementation decreases liver fat content in children with non-alcoholic fatty liver disease: Double-blind randomised controlled clinical trial. Arch. Dis. Child..

[108] Nobili V., Alisi A., Della Corte C., Risé P., Galli C., Agostoni C., Bedogni G. (2013). Docosahexaenoic acid for the treatment of fatty liver: Randomised controlled trial in children. Nutr. Metab. Cardiovasc. Dis..

[109] Nobili V., Carpino G., Alisi A., De Vito R., Franchitto A., Alpini G., Onori P., Gaudio E. (2014). Role of docosahexaenoic acid treatment in improving liver histology in pediatric nonalcoholic fatty liver disease. PLoS ONE.

[110] Boyraz M., Pirgon Ö., Dündar B., Çekmez F., Hatipoğlu N. (2015). Long-term treatment with n-3 polyunsaturated fatty acids as a monotherapy in children with nonalcoholic fatty liver disease. JCRPE J. Clin. Res. Pediatr. Endocrinol..

[111] Pacifico L., Bonci E., Di Martino M., Versacci P., Andreoli G., Silvestri L.M., Chiesa C. (2015). A double-blind, placebo-controlled randomized trial to evaluate the efficacy of docosahexaenoic acid supplementation on hepatic fat and associated cardiovascular risk factors in overweight children with nonalcoholic fatty liver disease. Nutr. Metab. Cardiovasc. Dis..

[112] Janczyk W., Lebensztejn D., Wierzbicka-Rucińska A., Mazur A., Neuhoff-Murawska J., Matusik P., Socha P. (2015). Omega-3 fatty acids therapy in children with nonalcoholic fatty liver disease: A randomized controlled trial. J. Pediatr..

[113] Spahis S., Alvarez F., Ahmed N., Dubois J., Jalbout R., Paganelli M., Grzywacz K., Delvin E., Peretti N., Levy E. (2018). Non-alcoholic fatty liver disease severity and metabolic complications in obese children: Impact of omega-3 fatty acids. J. Nutr. Biochem..

[114] Zöhrer E., Alisi A., Jahnel J., Mosca A., Della Corte C., Crudele A., Fauler G., Nobili V. (2017). Efficacy of docosahexaenoic acid–choline–vitamin E in paediatric NASH: A randomized controlled clinical trial. Appl. Physiol. Nutr. Metab..

[115] Spooner M.H., Jump D.B. (2019). Omega-3 fatty acids and nonalcoholic fatty liver disease in adults and children: Where do we stand?. Curr. Opin. Clin. Nutr. Metab. Care.

[116] Musa-Veloso K., Venditti C., Lee H.Y., Darch M., Floyd S., West S., Simon R. (2018). Systematic review and meta-analysis of controlled intervention studies on the effectiveness of long-chain omega-3 fatty acids in patients with nonalcoholic fatty liver disease. Nutr. Rev..

[117] Yu L., Yuan M., Wang L. (2017). The effect of omega-3 unsaturated fatty acids on non-alcoholic fatty liver disease: A systematic review and meta-analysis of RCTs. Pak. J. Med. Sci..

[118] Guo X.F., Yang B., Tang J., Li D. (2018). Fatty acid and non-alcoholic fatty liver disease: Meta-analyses of case-control and randomized controlled trials. Clin. Nutr..

[119] Day C.P., James O.F.W. (1998). Steatohepatitis: A tale of two “Hits”?. Gastroenterology.

[120] Couillard C., Ruel G., Archer W.R., Pomerleau S., Bergeron J., Couture P., Lamarche B., Bergeron N. (2005). Circulating levels of oxidative stress markers and endothelial adhesion molecules in men with abdominal obesity. J. Clin. Endocrinol. Metab..

[121] Masarone M., Rosato V., Dallio M., Gravina A.G., Aglitti A., Loguercio C., Federico A., Persico M. (2018). Role of oxidative stress in pathophysiology of nonalcoholic fatty liver disease. Oxid. Med. Cell. Longev..

[122] Tilg H., Moschen A.R. (2010). Evolution of inflammation in nonalcoholic fatty liver disease: The multiple parallel hits hypothesis. Hepatology.

[123] Marchesini G., Brizi M., Morselli-Labate A.M., Bianchi G., Bugianesi E., McCullough A.J., Forlani G., Melchionda N. (1999). Association of nonalcoholic fatty liver disease with insulin resistance. Am. J. Med..

[124] Palmieri V.O., Grattagliano I., Portincasa P., Palasciano G. (2006). Systemic oxidative alterations are associated with visceral adiposity and liver steatosis in patients with metabolic syndrome. J. Nutr..

[125] Lee G.Y., Han S.N. (2018). The role of vitamin E in immunity. Nutrients.

[126] Nagashimada M., Ota T. (2019). Role of vitamin E in nonalcoholic fatty liver disease. IUBMB Life.

[127] Sutti S., Jindal A., Locatelli I., Vacchiano M., Gigliotti L., Bozzola C., Albano E. (2014). Adaptive immune responses triggered by oxidative stress contribute to hepatic inflammation in NASH. Hepatology.

[128] Vos M.B., Colvin R., Belt P., Molleston J.P., Murray K.F., Rosenthal P., Schwimmer J.B., Tonascia J., Unalp A., Lavine J.E. (2012). Correlation of Vitamin E, uric acid and diet composition with histologic features of pediatric nonalcoholic fatty liver disease. J. Pediatr. Gastroenterol. Nutr..

[129] Vajro P., Mandato C., Franzese A., Ciccimarra E., Lucariello S., Savoia M., Capuano G., Migliaro F. (2004). Vitamin E treatment in pediatric obesity-related liver disease: A randomized study. J. Pediatr. Gastroenterol. Nutr..

[130] Nobili V., Manco M., Devito R., Ciampalini P., Piemonte F., Marcellini M. (2006). Effect of vitamin E on aminotransferase levels and insulin resistance in children with non-alcoholic fatty liver disease. Aliment. Pharmacol. Ther..

[131] Lavine J.E., Schwimmer J.B., Van Natta M.L., Molleston J.P., Murray K.F., Rosenthal P., Abrams S.H., Scheimann A.O., Sanyal A.J., Chalasani N. (2011). Effect of Vitamin e or metformin for treatment of nonalcoholic fatty liver disease in children and adolescents the tonic randomized controlled trial. JAMA J. Am. Med. Assoc..

[132] Cho T., Kim Y.J., Paik S.S. (2012). The efficacy of pharmacological treatment in pediatric nonalcoholic fatty liver disease. Pediatr. Gastroenterol. Hepatol. Nutr..

[133] Murer S.B., Aeberli I., Braegger C.P., Gittermann M., Hersberger M., Leonard S.W., Taylor A.W., Traber M.G., Zimmermann M.B. (2014). Antioxidant supplements reduced oxidative stress and stabilized liver function tests but did not reduce inflammation in a randomized controlled trial in obese children and adolescents. J. Nutr..

[134] Wang C.L., Liang L., Fu J.F., Zou C.C., Hong F., Xue J.Z., Lu J.R., Wu X.M. (2008). Effect of lifestyle intervention on non-alcoholic fatty liver disease in Chinese obese children. World J. Gastroenterol..

[135] Amanullah I., Khan Y.H., Anwar I., Gulzar A., Mallhi T.H., Raja A.A. (2019). Effect of Vitamin E in non-alcoholic fatty liver disease: A systematic review and meta-analysis of randomised controlled trials. Postgrad. Med. J..

[136] Klein E.A., Thompson I.M., Tangen C.M., Crowley J.J., Lucia S., Goodman P.J., Minasian L.M., Ford L.G., Parnes H.L., Gaziano J.M. (2011). Vitamin E and the risk of prostate cancer: The selenium and vitamin E cancer prevention trial (SELECT). JAMA J. Am. Med. Assoc..

[137] Bjelakovic G., Nikolova D., Gluud L.L., Simonetti R.G., Gluud C. (2008). Antioxidant supplements for prevention of mortality in healthy participants and patients with various diseases. Cochrane Database Syst. Rev..

[138] Bjelakovic G., Nikolova D., Gluud C. (2014). Antioxidant supplements and mortality. Curr. Opin. Clin. Nutr. Metab. Care.

[139] Chalasani N., Younossi Z., Lavine J.E., Charlton M., Cusi K., Rinella M., Harrison S.A., Brunt E.M., Sanyal A.J. (2018). The diagnosis and management of nonalcoholic fatty liver disease: Practice guidance from the American Association for the Study of Liver Diseases. Hepatology.

